# Immunomodulatory and cardio-protective effects of differentially originated multipotent mesenchymal stroma cells during polymicrobial sepsis in mice

**DOI:** 10.1007/s00068-025-02862-2

**Published:** 2025-04-20

**Authors:** Anna Kanewska, Ina Lackner, Anne Friedrich, Martina Winkelmann, Markus Rojewski, Birte Weber, Jochen Preßmar, Mario Perl, Hubert Schrezenmeier, Miriam Kalbitz

**Affiliations:** 1 Department of Trauma and Orthopedic Surgery, University Hospital Erlangen Friedrich-Alexander Universität Erlangen-Nürnberg (FAU), Erlangen, Germany; 2 Department of Traumatology, Hand, Plastic and Reconstructive Surgery, Center of Surgery, University of Ulm, Ulm, Germany; 3 Institute for Transfusion Medicine, University Medical Center Ulm, Ulm, Germany; 4 Institute for Clinical Transfusion Medicine and Immunogenetics Ulm, German Red Cross Blood Donation Service, Ulm, Germany; 5 Department of Trauma Surgery and Orthopedics, University Hospital Frankfurt, Goethe-University, Frankfurt Am Main, Germany; 6 Military Medical City Hospital (MMCH), Doha, Qatar

**Keywords:** Septic cardiomyopathy, CLP sepsis, Cardiac dysfunction, ASC treatment, BMMSC treatment, Immunotherapy, Tissue regeneration

## Abstract

**Purpose:**

Sepsis is a life-threatening condition with cardiac complications being an independent predictor of poor outcome. Although their mechanisms have been widely investigated, therapeutic options remain limited. One promising therapeutic tool are mesenchymal stromal cells (MSCs). The aim of this study is to investigate the immunomodulatory effects of human MSCs from two different sources (bone marrow/BMMSC and adipose tissue/ASC) and to evaluate their cardioprotective potential.

**Methods:**

60 adult male C57BL/6 mice were divided into sham, sepsis (cecal ligation puncture (CLP)) and two i.v. treatment groups CLP + human BMMSC and CLP + human ASC with 5 animals in each group. The observation periods were 8, 24 and 72 h. Left ventricular tissue was analyzed histologically, by qPCR (*C3ar*, *C5ar1*, *Il-1b*, *Il-6*, *Il-10*, *Tlr2*, *Tlr4*, *Tnfa*, and *Nlrp3*) and western blot. Cardiac damage markers troponin I and heart fatty acid binding protein (HFABP) were detected in serum by ELISA.

**Results:**

Troponin I and HFABP were significantly increased in CLP group after 8 h compared to sham. In cardiac tissue the expression of *C3ar*, *C5ar1*, *Il-1b*, *Il-6*, *Il-10*, *Tlr2*, *Tlr4*, *Tnfa* and *Nlrp3* inflammasome was upregulated up to 24h after CLP compared to sham. After BMMSC treatment, *C3ar* as well as *C5ar*, *Tlr2* and *Il-10* mRNA expression in left ventricle was downregulated compared to CLP, whereas ASC treatment was associated with the downregulation of *Il-6* and *Nlrp3*.

**Conclusions:**

CLP-induced polymicrobial sepsis in mice was associated with cardiac damage and increased inflammation in left ventricular tissue. Therapeutic systemic application of human BMMSC and ASC ameliorated damage and inflammation in the heart.

**Supplementary Information:**

The online version contains supplementary material available at 10.1007/s00068-025-02862-2.

## Introduction

Sepsis is a life-threatening condition caused by a dysregulated host response to infection leading to organ dysfunction such as septic cardiomyopathy, acute kidney injury, or acute respiratory distress syndrome [1]. With an intensive care unit (ICU) prevalence of 30% and lethality of 47% [2], sepsis remains a challenge to the healthcare system [3, 4].

In septic patients, myocardial dysfunction in particular increases the mortality by up to 70–90% [5, 6]. Septic cardiomyopathy is described as a global systolic- and diastolic dysfunction, including left- and right ventricular malfunction [7], affecting up to 80% of patients with septic shock [8]. Sepsis-induced organ failure, like cardiac dysfunction has been shown to be associated with systemically elevated pro-inflammatory mediators, cytokines and complement activation [9]. Levels of systemically elevated pro-inflammatory cytokines such as interleukin (IL)−6, IL-1beta and the tumor necrosis factor (TNF) correlated with sepsis severity and organ dysfunction [10]. In particular, the development of septic cardiomyopathy has been shown to be mediated by complement component receptors 3a (C3aR) and C5aR1/2 [11]. Activation of pattern-recognition receptors such as toll-like receptors (TLR), Nucleotide oligomerization domain (NOD)- leucine rich-repeat (LRR) and pyrin domain-containing protein 3 (NLRP3) inflammasome, have been associated with cardiac dysfunction in cecal ligation and puncture (CLP)-induced sepsis models in vivo [12–14]. In addition, the inflammatory response can be exacerbated by additional bacterial endotoxins such as lipopolysaccharides (LPS) [15]. Notably, the overwhelming inflammatory response leading to septic cardiomyopathy can also be induced by damage-associated molecular patterns (DAMPs) released upon tissue damage, such as extracellular histones and the high-mobility group box 1 (HMGB1) protein [16, 17].

Although there is a growing understanding of the inflammatory mechanisms underlying septic cardiomyopathy, little is known about the resulting therapeutic options.

To date, therapeutic approaches to treat septic cardiac dysfunction focus on symptomatic treatment and antibiotics [3, 18]. Recently, several therapeutic concepts have been described to address the inflammatory response during sepsis, including the administration of corticosteroids, cytokine neutralizing agents, hemadsorption and immunostimulants [18]. While immunomodulatory approaches seemed to be promising, the results in the phase III and IV trials were rather disappointing with only small clinical improvements observed [18]. Possible reasons may be the difficulty of addressing the heterogeneity of the different causes of sepsis and its underlying mechanisms [18, 19].

During the last decade, the application of multipotent mesenchymal stromal cells (MSCs) has become an auspicious candidate for the regeneration of damaged tissues, in general [20]. In response to DAMPs as chemoattractant, MSCs are able to specifically migrate to injured tissue and to establish a pro-regenerative microenvironment [21]. MSCs have been shown to have immunomodulatory effects in various tissues like colon, liver, and lung during CLP sepsis [22–24]. MSCs can respond to inflammatory signals and to DAMPs, i.e. they are sensors for a disturbed environment [25–27].

Furthermore, in case of murine myocardial ischemia/reperfusion injury and in rat with acute myocardial infarction, regenerative effects of MSCs on the myocardium have been described: the infarct size was reduced and the cardiac systolic and diastolic function was preserved via MSC-derived exosomes [28, 29]. Therefore, MSCs may be a promising therapeutic candidate for the treatment of sepsis-induced cardiac dysfunction. The aim of the present study was to investigate the therapeutic use of MSCs in septic cardiomyopathy by focusing on the immunomodulatory effects of these cells. Another aspect addressed in the study is the importance of the MSCs source for possible effects on the myocardium. While previous studies on cardioprotective effects of MSCs focused on bone marrow-derived MSCs [28, 30], MSCs can be also isolated from different tissue sources such as adipose tissue, umbilical cord, and even menstrual blood [31]. Some experimental studies of systemic inflammation during sepsis showed that the application of BMMSCs is preferable due to a higher expression of the soluble cytokine receptors like soluble tumor necrosis factor receptor (sTNFR1) and soluble vascular endothelial growth factor receptor-1 (sVEGFR1) [32], while other studies indicated the beneficial effects of ASCs due to their easier availability [33] or showed no differences between BMMSCs on systemic inflammation during sepsis [34]. However, neither a consistent source for MSC therapy in sepsis has been achieved nor different sources have been evaluated for their therapeutic potential in septic cardiomyopathy.

Therefore, we addressed the questions of whether MSCs have cardioprotective effect in CLP sepsis and further, which source of MSCs should be preferred, BMMSCs or ASCs.

## Material and methods

### Manufacturing of BMMSC and ASC

Bone aspirate and lipoaspirate of volunteer donors were obtained after informed consent. (approved by Ethics Committee of the University of Ulm, Germany, procedures performed in accordance with the Declaration of Helsinki). From this starting material BMMSCs and ASCs were manufactured according to Good Manufacturing Practice (GMP certificate number DE_BW_01_GMP_2020_0042) using aseptic procedures and disposable sterile single-use equipment for all product contact steps.

BMMSCs were generated from a 25 ml- 35 ml iliac crest bone marrow aspirate from healthy volunteer bone marrow donors (accessed 30/07/2012, 17/09/2012). BMMSC isolation and expansion were performed according to the previously described standard operating procedures [35]. Briefly, the bone marrow cells were seeded at a density of 50,000 white blood cells (WBC) per cm.^2^ on 1- or 2-chamber CellSTACKs (Corning, USA) in alphaMEM (Lonza, Switzerland), supplemented with 5% platelet lysate (PL) and 1 I.U. heparin/ml final concentration (PL from IKT Ulm, Germany) for isolation and the first expansion step and with 8% PL and 1 I.U. heparin/ml final concentration for higher passages. The product consisted of fresh, bone marrow MSCs, expressing the identity markers CD90, CD73, CD105 and lacking expression of impurity markers CD3, CD14, CD34, CD45 and HLA class II, with a 90% viability rate [35]. Such BMMSCs of passage 1 produced in this GMP-compliant manner have already been used in clinical trials: (i) EudraCT No.: 2011–005441-13, where signs of bone regeneration of non-union fractures were described [36], (ii) EudraCT No.: 2012–002010-39, where the therapy of femoral head necrosis was evaluated as successful [37] (iii) EudraCT No. 2012–003139-50, where the therapy showed augmented regeneration of mandibular bone [38]. The BMMSCs were well tolerated in all of these studies and no serious adverse events were reported which were attributed to the BMMSC treatment [36–38]

In the herby presented study, 1 × 10^6^ cells were conditioned as described below and filled in a 1 ml syringe for injection into each animal.

ASCs were generated from approximately 60 g of sub-cutaneous human adipose tissue from healthy volunteers (accessed: 08/09/2015, 01/12/2015) each. Adipose tissue was obtained from the volunteers via liposuction. Aliquots of 20 ml – 25 ml of tissue were generated by digestion with GMP-grade collagenase (Nordmark, Germany), mixed with DPBS and centrifuged at 340 g for 4 min. The upper (oil and mature adipocytes) and lower (blood and fluid) phases were discarded. The stroma vascular fraction (SVF) was obtained from the remaining tissue by collagenase digestion (± 14 PZ-units of collagenase/10 g of pure adipose tissue; according to Wünsch [39]) at 37 °C for 45 min. The digestion was stopped by the addition of alphaMEM, supplemented with 5% PL and 1 I.U./ml heparin. After homogenization of the solution by pipetting, the suspension was passed through a 100 μm cell strainer. The SVF was collected by centrifugation (20 °C, 10 min, 600 × g) and resuspended in media. SVF was seeded at a density of 4,000 cells/cm^2^ in 2-chamber CellSTACKs in alphaMEM, supplemented with 5% PL and 1 I.U. heparin/ml. Media change was performed after 4 and 6 days. ASC of passage 0 were harvested after 8 days using TrypZean as detachment reagent. The product consist of fresh, adipose derived mesenchymal stromal cells (ASC), expressing the identity markers CD13, CD90, CD73, CD105 and lacking expression of impurity markers CD3, CD14, CD34, CD45 and HLA class II, with a 90% viability rate [35]. ASCs of passage 1 produced according to this GMP-compliant protocol have already been used in the clinical trial ADIPOA2 (EudraCT No.: 2015–002125-19) for the treatment of mild to moderate arthritis (Kallgren-Lawrence-Scale grade 2 or 3) (manuscript in preparation). Further passaging for this study was performed by seeding the ASC on 2-chamber CellSTACKs at a density of 2,000 cells/cm^2^ for passage 1 and 1,000 to 2,000 cells for passages ≥ 1 in alphaMEM, supplemented with 5% PL and 1 I.U. heparin/ml for another 4—6 days.

It has also been confirmed in previous experiments [40] that MSCs which were isolated, expanded, cryopreserved, thawed and further expanded in other settings after a long-term storage time remained functional in immunomodulation.

### Preparation of BMMSC and ASC injection

For injections, only BMMSCs and ASCs of passages ≤ 3 were used. Cells were detached for harvesting via TrypZean. Cell count was determined by trypan blue (Merck, Germany) staining in a calibrated Neubauer improved chamber. Immediately before application, cells were resuspended and incubated in 1 ml of physiological saline solution supplemented with 5000 I.U. heparin/ml at 20° C for 30 min to prevent aggregation, rinsed once with Dulbecco’s phosphate buffered saline without Ca^2+^/Mg^2^ (DPBS), centrifuged at 400 × g at 20 °C for 5 min, and the supernatant was discarded. Individual doses of 1 × 10^6^ cells each (BMMSC and ASC) were resuspended in 250 µl DPBS, passed through a 100 µm strainer and transferred in 1 ml syringes and injected into animals.

### Animal experiments

All animal experiments were approved by the local animal welfare committee (Regierungspräsidium Tübingen, no. 1255) and this study was performed in accordance with relevant guidelines and regulations. All methods are reported in accordance with ARRIVE guidelines. For the animal experiments, 60 male C57BL/6 mice (Jackson Laboratory) were used with an age between 10–12 weeks and a weight between 25–30 g. Littermate mice were housed in groups of up to five animals with free access to food and wate*r*.

Mice were randomly assigned to the following treatment groups: sham, CLP, CLP + BMMSC and CLP + ASC with n = 5 animals in each group. The observation time points included 8, 24 and 72 h. Mice were sacrificed at the end of each observation time point.

### Surgical procedure

All surgical procedures were conducted under general anesthesia and analgesia. For analgesia, mice received 0.05 mg/kg buprenorphine subcutaneously (s.c.) during surgery and post-surgery every six hours. For anesthesia, mice received a mixture of 12.5 mg/ml (80–100 mg per kg of body weight) ketamine and 2.5 mg/ml (5–15 mg per kg of body weight) xylazine intraperitoneally (i.p.). Polymicrobial sepsis was induced by CLP as previously described by Rittirsch et al. [41]. Briefly, an abdominal midline incision was performed at the shaved abdominal region. A midgrade polymicrobial sepsis was induced by ligating the cecum halfway between the ileocecal valve and the ending of the cecum. By using a 22G needle, a puncture of the cecum was made. Following, a minimal amount of bowel content was then extruded, and the cecum was repositioned. The abdominal incision was closed in layers. Fluid resuscitation was performed by applying 1 ml of 0.9% saline s.c.. The sham control animals also received analgesia, anesthesia, abdominal shaving and fluid resuscitation but no surgical procedures. After fluid resuscitation, the CLP mice were randomly assigned into 3 treatment arms: PBS, BMMSC or ASC. Subsequently, the mice received either an equivalent amount (250 μl) of sterile phosphate buffered saline (PBS), 1 × 10^6^ BMMSCs (in 250 μl PBS) or 1 × 10^6^ cells ASCs (in 250 μl PBS), which were applied intravenously (i.v.) in the tail vein using a 30G cannula. After the surgical procedure, mice were allowed to move freely with access to food and water ad libitum. The mice were closely monitored during the observation period of 8, 24 and 72 h and 0.05 mg/kg buprenorphine was applied every 6 h s.c. for adequate pain medication. In addition, fluid resuscitation was secured by applying 1 ml of 0.9% saline s.c. every 24 h.

### Sample collection

After the follow-up period of either 8, 24 or 72 h, the mice were euthanized. Blood was taken immediately after euthanasia by cardiac puncture. Plasma samples were collected after centrifugation for 5 ​min (800 × *g*, 4 ​°C) and a second centrifugation for 2 ​min (13,000 × *g*, 4 ​°C). The plasma samples were stored at − 80 ​°C until analysis. Samples of the cardiac left ventricle were taken and either quick-frozen by liquid nitrogen or fixed in 4% paraformaldehyde (PFA) for 48 ​h for histology (supplemental Fig. 1).

**Fig. 1:**
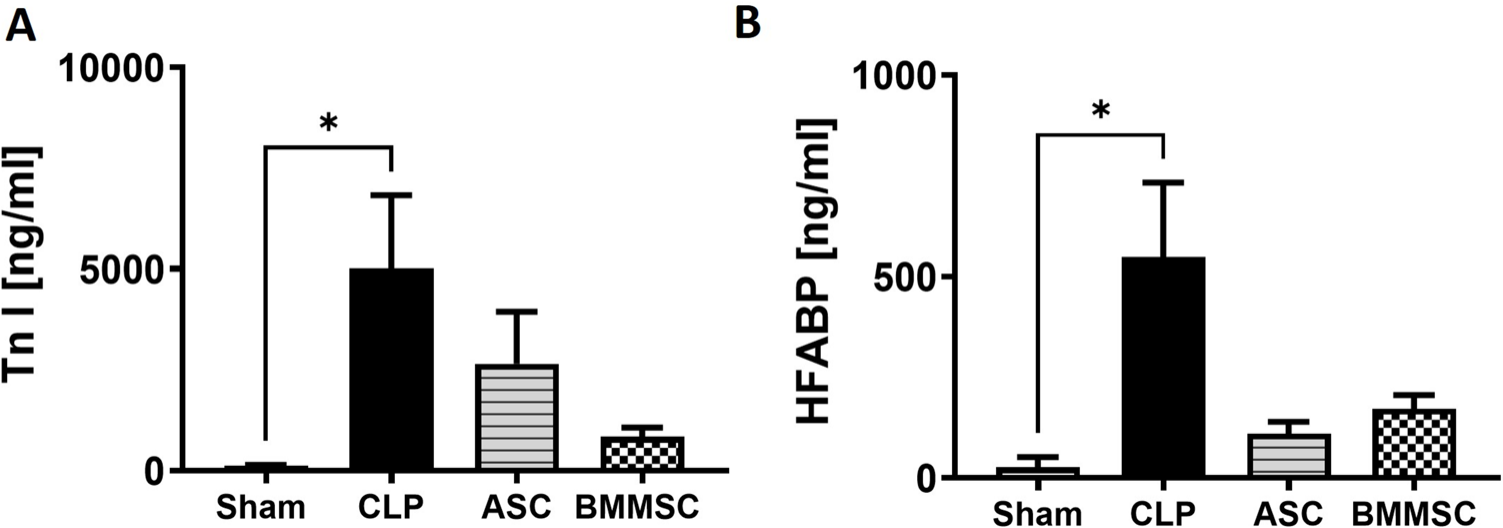
Elevated plasma levels of Troponin I and Heart-Type Fatty Acid Binding Protein (HFABP) in cecal ligation puncture procedure (CLP) mice in comparison to sham. Wildtype mice received either sham treatment, CLP procedure with or without Bone-marrow derived mesenchymal stem cells (BMMSC) or Adipose Stromal Cells (ASC) therapy. Plasma of 8 h following CLP procedure was used for ELISA analysis. **A** Plasma Troponin I level in ng/ml. **B** Plasma HFABP level in ng/ml. Data are presented as mean ± SEM. p ≤ 0.05 was considered as statistically significant. *p ≤ 0.05, sham vs. CLP, sham vs. ASC, sham vs. BMMSC. Each bar N = 5

### Troponin/HFABP ELISA

To measure the cardiac damage systemically, Troponin I concentration was quantified via Mouse TNNI3/Cardiac Troponin I Sandwich ELISA (Lifespan Biosciences, Lynnwood, Washington, USA; LS-F24180-1) and heart fatty acid binding protein (HFABP) concentration was measured via the mouse cardiac FABP ELISA (Life Diagnostics, West Chester, PA, USA; Cat. No. HFABP-1) in EDTA plasma according to the manufacturer’s instructions.

### RNA isolation and RT-qPCR

Collected tissue from left ventricle was homogenized with 0.5 ml Precellys® Lysing Kit 1.4 mm ceramic beads and Precellys®24 tissue homogenizer (Bertin Technologies, Montigny-le-Bretonneux, France) and RNA was isolated using AllPrep® DNA/RNA/miRNA Universal Kit (Qiagen, Hilden, Germany). The respective RNA samples were reverse transcribed into cDNA using SuperScript™ IV VILO™ MasterMix with ezDNAse (Invitrogen, Carlsbad, CA, USA). For quantitative PCR the PowerUp™ SYBR™ Green Master Mix (Applied Biosystems, Waltham, MA, USA) was used and the QuantStudio3 system (Applied Biosystems, Waltham, MA, USA) was utilized. All procedures were performed according to the manufacturer’s instructions. Quantitative mRNA expression of murine C3a receptor (*C3ar*), C5a receptor 1 (*C5ar1*), interleukin (*Il*)-*1b*, *Il-6*, *Il-10*, NLR family pyrin domain containing 3 (*Nlrp3*), toll-like receptor (*Tlr*)2, *Tlr4*, *Tlr9*, tumor necrosis factor (*Tnfa*) and Troponin I (*TnI*) was examined. For normalization, glutaraldehyde-phosphate dehydrogenase (*Gapdh*) was used as housekeeping gene and analysis was made with the cycle threshold method (ΔΔCt). Results are presented as mean fold change.

### Protein isolation and gel electrophoresis

For protein isolation, left ventricular tissue was homogenized in RIPA Lysis and Extraction Buffer (ThermoFisher Scientific, Waltham, MA, USA), containing phenylmethanesulfonylfluoride fluoride (ThermoFisher Scientific, Waltham, MA, USA), protease Inhibitor Cocktail (Sigma-Aldrich, St. Louis, MO, USA) and sodium orthovanadate (Sigma-Aldrich, St. Louis, MO, USA) using 2 ml Precellys® Lysing Kit 1.4 mm ceramic beads and Precellys®24 Tissue Homogenizer (Bertin Technologies, Montigny-le-Bretonneux, France). Total protein concentration was measured using Pierce™ BCA Protein Assay Kit (ThermoFisher Scientific, Waltham, MA, USA) and 15 µg protein equivalents of tissue homogenates were loaded onto a 7–10% TGX Stain-Free™ FastCast™ acrylamide gel (BIO-RAD, Hercules, CA, USA) for gel electrophoresis.

### Western blot

After gel electrophoresis, the samples were transferred from 7–10% acrylamide gel onto Mini PVDF Membrane (BIO-RAD, Hercules, CA, USA) by using the Trans-Blot® Turbo™ Transfer System (BIO-RAD, Hercules, CA, USA). Membranes were blocked with 5% dry milk for 1.5 h at room temperature and then incubated with the primary antibodies against C3 (abcam, Cambridge, UK, [EPR19394] ab200999, 1:1000), NLRP3 (abcam, Cambridge, UK, [EPR23094-1] ab263899, 1:500) or myeloid differentiation primary-response protein 88 (MyD88) (abcam, Cambridge, UK, ab219413, 1:1000; 5% bovine serum albumen (BSA) was used for blocking) overnight at 4 °C. After washing, the membrane was incubated with anti-rabbit IgG HRP-linked antibody (CellSignaling Technology, Danvers, MA, USA) for 1 h at room temperature. Signal development was analyzed by ChemiDoc MP Imaging System (BIO-RAD, Hercules, CA, USA). Protein expression was quantified with the ImageLab Software (6.1.0. build 7, BIO-RAD, Hercules, CA, USA). Protein expression was normalized to the total l protein. Results are expressed as mean pixel density. Western blot analysis of other cytokines did not show any clear bands or significant differences between the groups, therefore that data is not shown.

### Histological analysis -hematoxylin and eosin staining (HE staining)

Formalin-fixed and paraffin-embedded tissue sections from left ventricles were used. Tissue sections were deparaffinized rehydrated and stained with hematoxylin and eosin staining kit (Morphisto, Frankfurt am Main, Germany) according to the manufacturer’s protocol. To quantification of myocardial damage, a heart injury score was defined as described previously [42, 43]. For determination of the heart injury score, the HE-stained sections of left ventricle were scored by two independent observers for 1) apoptosis, 2) contraction band necrosis, 3) neutrophil infiltration, 4) intramuscular hemorrhage, 5) rupture, 6) edema and 7) ischemia. In each section, three representative, non-marginal sections were selected at × 40 magnification (Axio observer 7, ZEN 3.4, Fa. Carl Zeiss, Jena, Germany) and the mean of the subsumed score calculated.

### Statistical analysis

Statistical analysis was performed with GraphPad Prism 10.1.2 software (GraphPad Software, Inc., San Diego, CA, USA). Normality analysis was performed with the Shapiro–Wilk-Test. Data, which passed the normality analysis, was analyzed by one-way Analysis of Variance (ANOVA), followed by Dunnett’s multiple comparison test. Data, which did not pass the normality test, were analyzed by Kruskal–Wallis ANOVA, followed by Dunn’s multiple comparison test. Unless stated otherwise, the CLP mice without additional MSC treatment were used as control. If both BMMSC and ASC treated mice showed significant changes in comparison to the CLP mice without additional MSC treatment, those two groups were additionally analyzed by unpaired, two-tailored T-Test. All values are expressed as mean ± SEM. p ≤ 0.05 was considered as statistically significant.

## Results

### Succeeded in vivo model for polymicrobial sepsis but no changes in murine left ventricle through MSC application

**Fig. 2:**
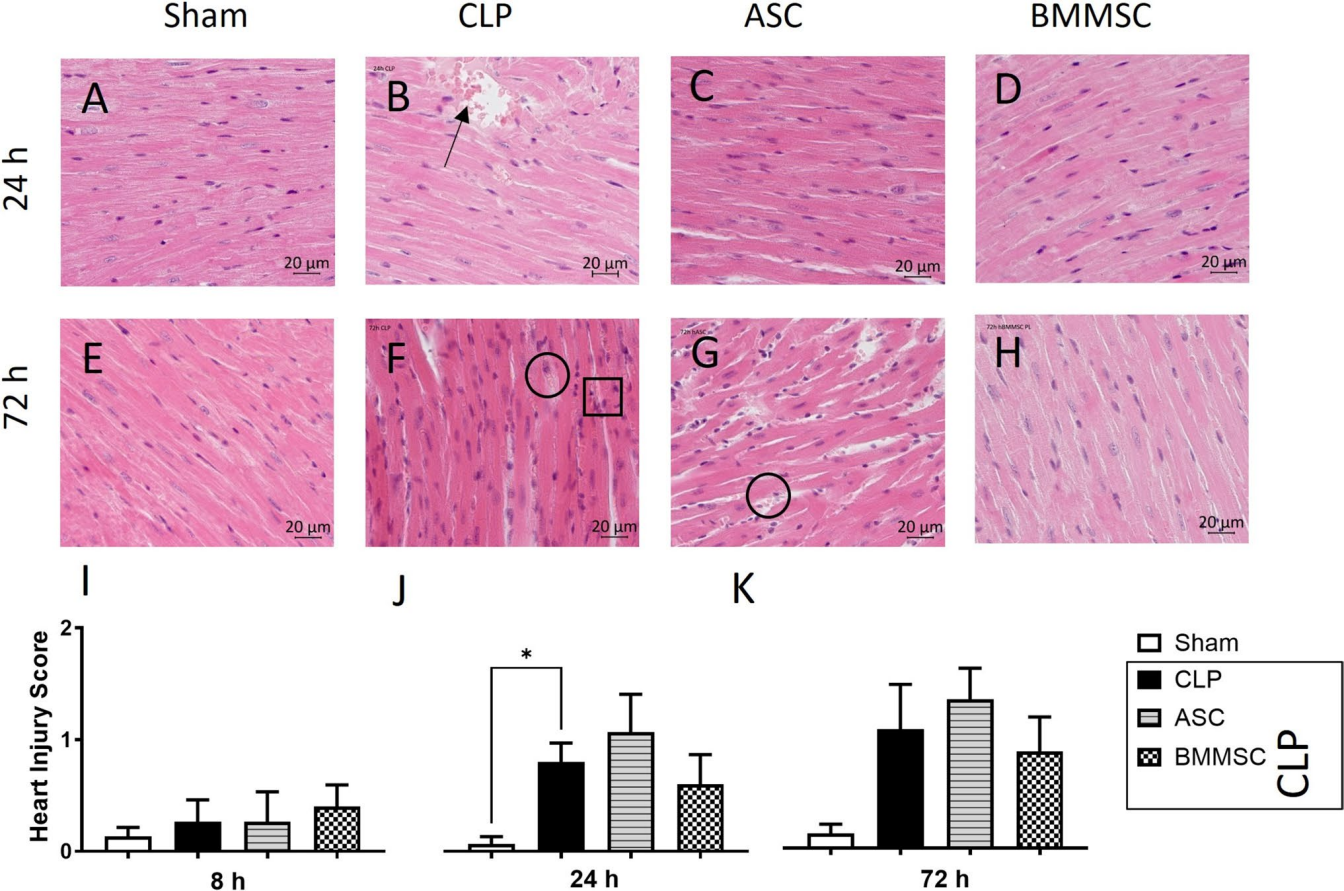
Morphological changes in all CLP treatment groups in comparison to sham. Wildtype mice received either sham treatment, CLP procedure with or without BMMSC or ACS therapy. Left ventricles 8 h, 24 h or 72 h following CLP procedure were processed into paraffine slides and stained with H.E. **A–H** Representative images of Hematoxylin Eosin staining of the left ventricle of mice sacrificed after 24 h (**A–D**) and 72 h (**E–H**) belonging to treatment groups sham, CLP, BMMSC and ACS with ventricle rupture (**B**, black arrow), apoptosis (**F**, black square) and intramuscular bleeding (**F**, **H**, black circle). **I** Comparison of the histology damage scores between the different treatment groups sacrificed after 8 h, 24 h and 72 h. Data are presented as mean ± SEM. p ≤ 0.05 was considered as statistically significant. *p ≤ 0.05, sham vs. CLP, sham vs. ASC, sham vs. BMMSC. Each bar N = 5

Using a murine model of polymicrobial sepsis, we analyzed systemic markers of cardiac damage and histomorphological pathologies in the left ventricle. Troponin I and HFABP were detected by ELISA early after CLP. Both markers significantly increased 8 h after CLP in mice as compared to the sham (Fig. 1 A, B), however no significant differences in Troponin I or HFABP levels (Fig. 1 A, B) could be observed in any MSC groups. Despite this, systemic Troponin I levels showed a declining trend following BMMSC administration. Histological scores of murine left ventricles were not significantly different between CLP mice, BMMSC mice, or ASC mice (Fig. 2).

For the analysis of local cardiac damage, HE-stained tissue sections of the left ventricle were scored for damage as previously described **(**Fig. 2**).** 8 h after CLP, there were no significant differences in cardiac damage scores between the respective groups **(**Fig. 2I**).** As compared to sham mice, animals that underwent the CLP procedure (CLP, ASC, and BMMSC) showed slightly higher cardiac injury scores in their left ventricle 24 h and 72 h after CLP. It was found that apoptotic cell nuclei, contraction band necrosis, intramuscular hemorrhage (Fig. 2 B), and ventricular tissue rupture were more prevalent 24 h after CLP.

### Elevated *Tnfa* expression in the left ventricle of CLP mice

**Fig. 3:**
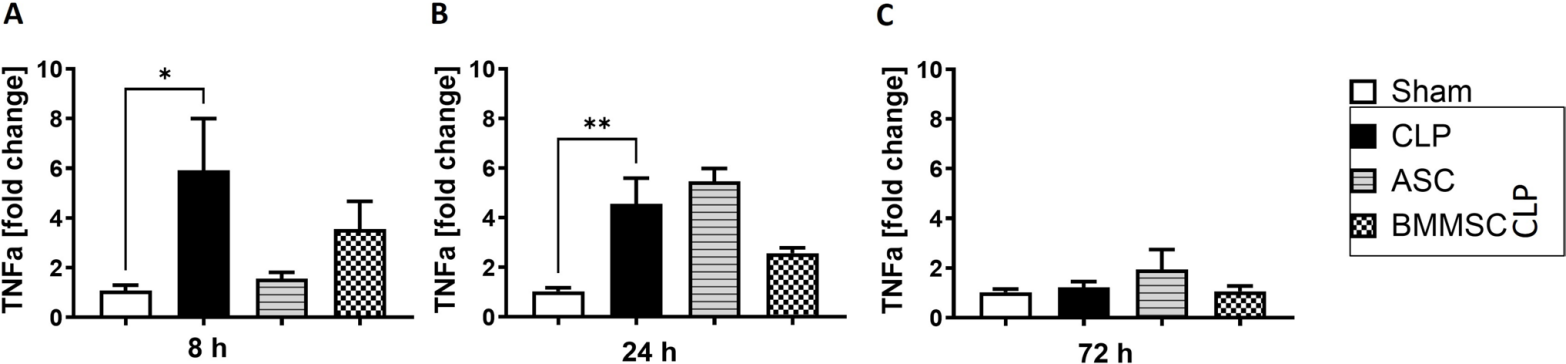
Upregulated tumor necrosis factor alpha (*Tnfa*) gene expression in left ventricle of CLP mice. Wildtype mice received either sham treatment, CLP procedure with or without BMMSC or ACS therapy. Left ventricle was analyzed 8 h, 24 h or 72 h following CLP procedure. **A–C** mRNA expression of tumor necrosis factor in fold change. Data are presented as mean ± SEM. p ≤ 0.05 was considered as statistically significant. *p ≤ 0.05, ** p ≤ 0.005, CLP vs. sham, CLP vs. BMMSC, CLP vs. ASC. Each bar N = 5

As part of the subsequent analysis of local inflammation in murine left ventricles, we observed an upregulation of *Tnfa* 8 h and 24 h following CLP in comparison with the sham group (Fig. 3 A, B). There was no evidence of elevated *Tnfa* levels in CLP mice 72 h after the procedure. Neither BMMSC nor ASC seemed to have a significant effect on *Tnfa* expression (Fig. 3 A-C).

### Upregulation of *C3ar *and* C5ar1 *in CLP and downregulation of *C3ar* in BMMSC

**Fig. 4:**
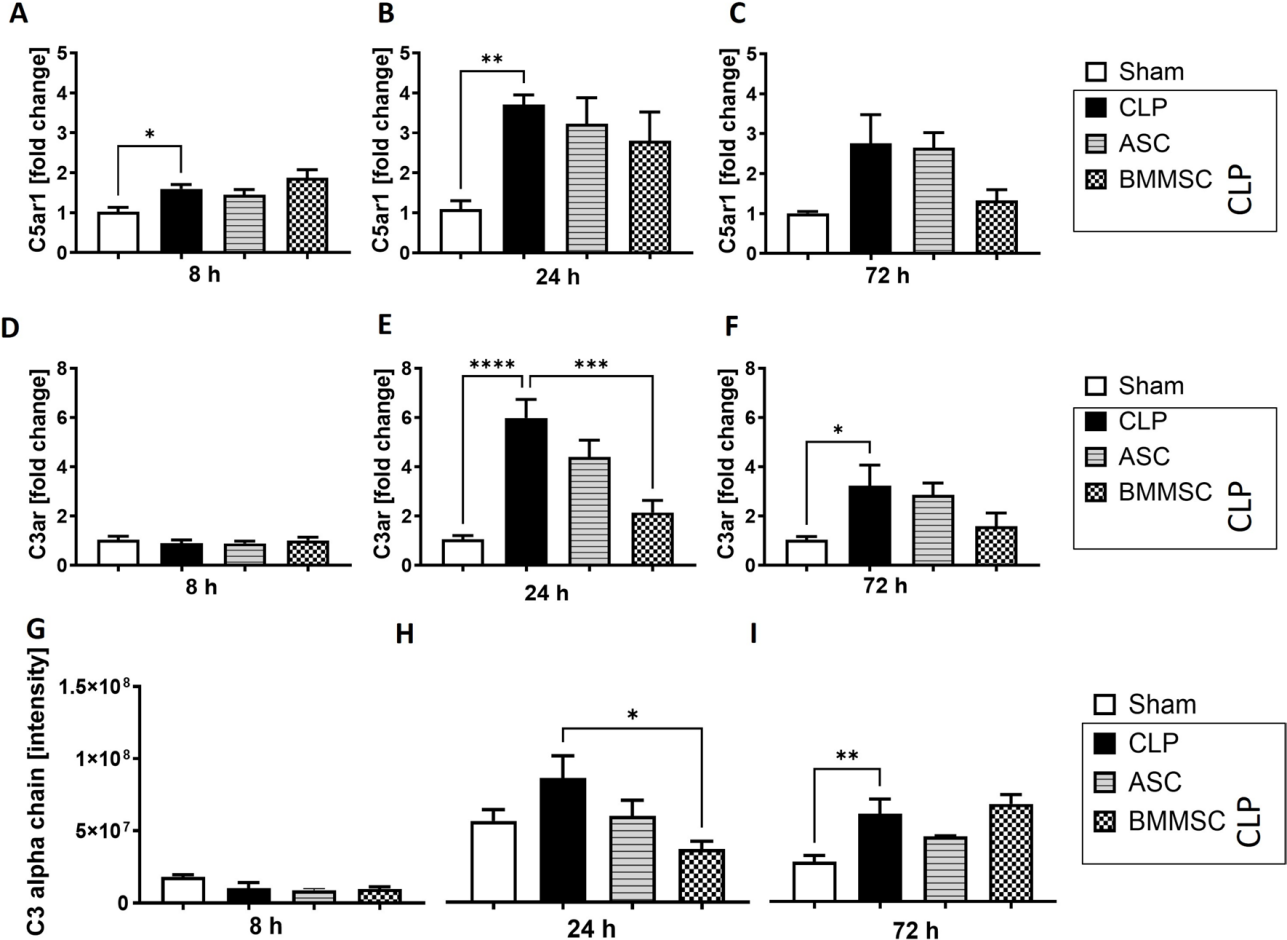
Complement component 3a (*C3ar*) and complement 5a receptor 1 (*C5ar1*) upregulation in left ventricle in polymicrobial sepsis. Wildtype mice received either sham treatment, CLP procedure with or without BMMSC or ACS therapy. Left ventricle was analyzed 8 h, 24 h or 72 h after CLP + treatment. **A–C** Local mRNA expression of *C5ar1* in fold change. **D–F** mRNA expression of *C3ar* in fold change. **G–I** Protein expression of C3 alpha chain in protein band intensity. Data are presented as mean ± SEM. p ≤ 0.05 was considered as statistically significant. *p ≤ 0.05, ** p ≤ 0.005, *** p ≤ 0.0005, CLP vs. sham, CLP vs. BMMSC, CLP vs. ASC. Each bar N = 5

We further investigated the complement response in the left ventricle of mice after CLP. Mice with polymicrobial sepsis exhibited significant upregulation of *C5ar1* gene expression after 8 and 24 h, respectively (Fig. 4 A, B). This was accompanied by the significant upregulation of *C3ar* (Fig. 4 E, F) gene expression 24 h and 72 h after CLP and protein expression after 72 h (Fig. 4I). Neither C5ar1 gene expression at 72 h nor C3ar gene expression at 8 h differed significantly from sham group.

BMMSC mice on the other hand displayed significantly lower levels of gene and protein expression of *C3aR* in left ventricle at 24 h than CLP mice (Fig. 4E, H. No changes after BMMSC treatment were observed for C3a (Fig. 4) at 8 h or 72 h. Neither C3ar nor C5ar1 were affected by ASCs (Fig. 4).

### Downregulation of* Il-6* and *Il-10* in left ventricle of CLP mice after MSC administration

**Fig. 5:**
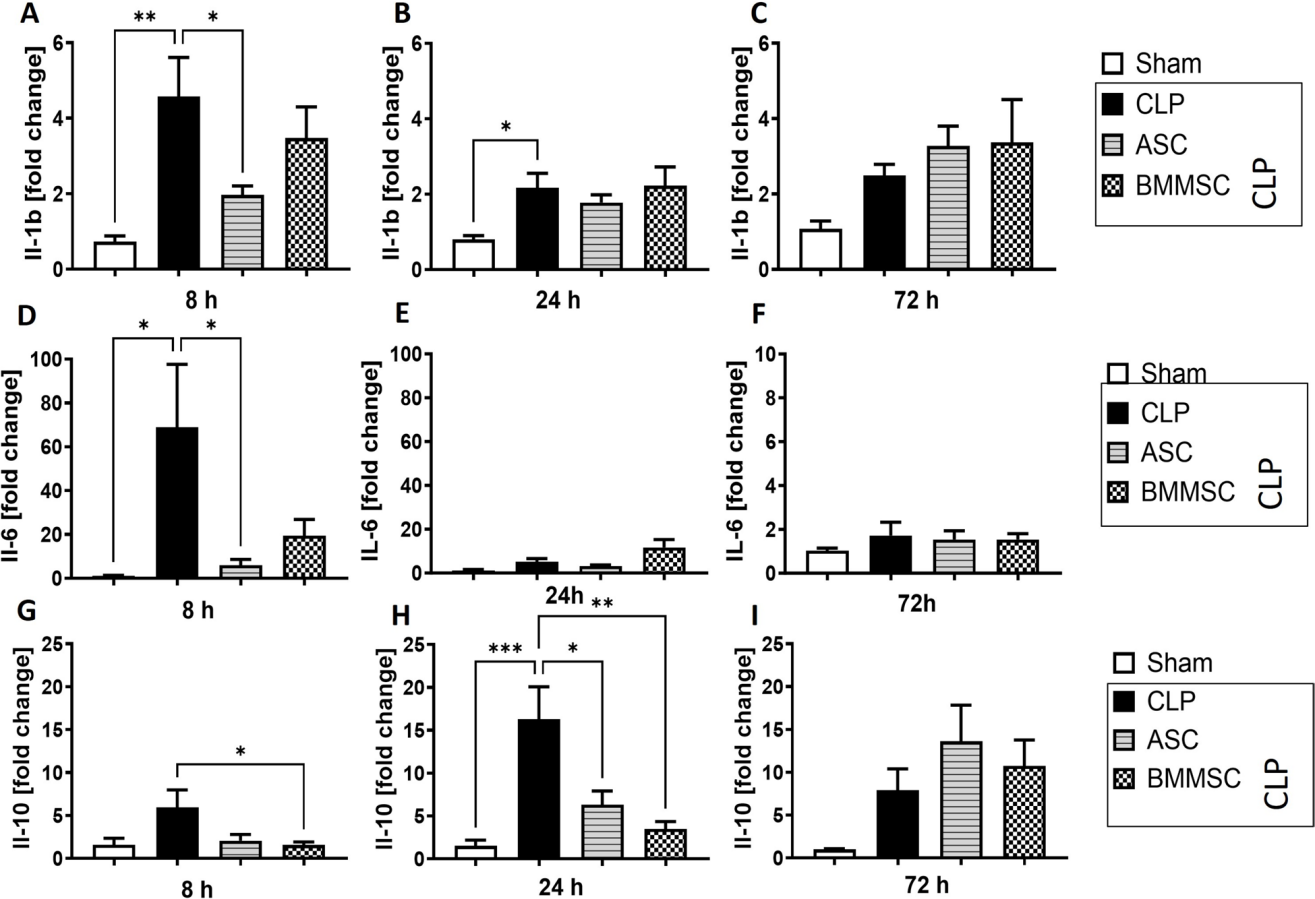
Interleukin (*Il)−6*, *Il-1b* and *Il-10* upregulation in left ventricle of CLP mice. Wildtype mice received either sham treatment, CLP procedure with or without BMMSC or ACS therapy. Left ventricle was analyzed 8 h, 24 h or 72 h following CLP procedure. **A–C** mRNA expression of *Il-1b* in fold change. **D–F** mRNA expression of *Il-6* in fold change. **G–I** mRNA expression of *Il-10* in fold change. Data are presented as mean ± SEM. p ≤ 0.05 was considered as statistically significant. *p ≤ 0.05, ** p ≤ 0.005, *** p ≤ 0.0005, CLP vs. sham, CLP vs. BMMSC, CLP vs. ASC. Each bar N = 5

We also observed an elevation of all three interleukins (IL-6, IL-1β and IL-10) in left ventricle of mice 8 h after CLP treatment (Fig. 5 A, D, G). Moreover, *Il-1β* and *Il-10* gene expression in left ventricle remained significantly increased 24 h after CLP onset (Fig. 5B, H). A 72 h comparison of CLP versus sham group did not show any statistically significant difference between the two groups.

After MSC application the increase of *Il-6* and *Il-10* was removed (Fig. 5D, G), more precisely BMMSC administration caused a downregulation of *Il-6* while ASC treatment did for *Il-10*. A reduced expression of *Il-10* was seen again in the BMMSC and ASC groups after 24 h, but not at 72 h (Fig. 5H, I). Apart from the significant changes in *Il-6* and *Il-10* expression, no significant effect of MSC on the *Il-1β* expression in CLP-mice occurred.

### Downregulation of *Tlr2* in murine left ventricle after MSC administration in CLP mice

**Fig. 6:**
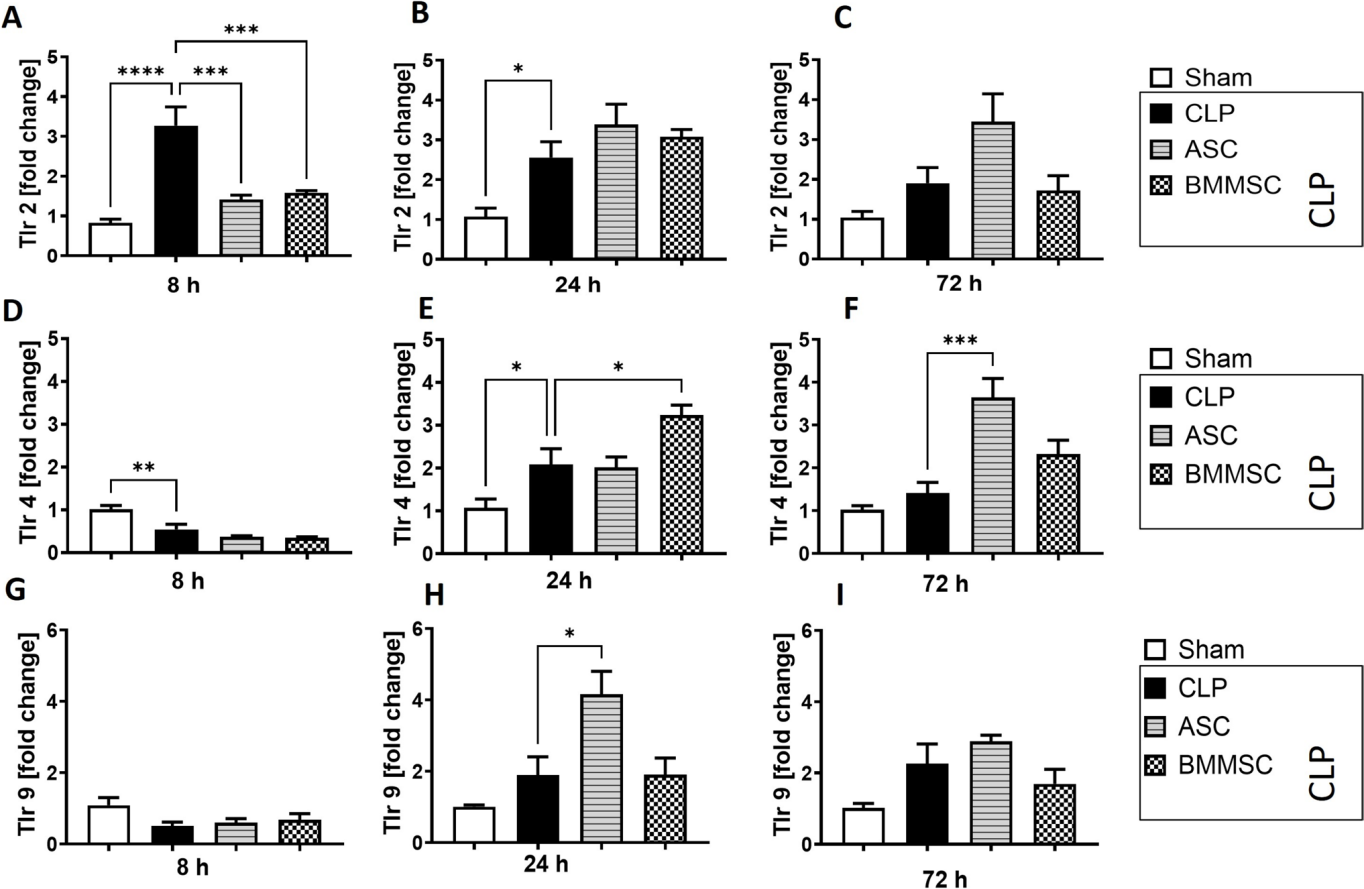
Upregulation of Toll-Like Receptor (*Tlr*) 2 in left ventricle in polymicrobial sepsis. Wildtype mice received either sham treatment, CLP procedure with or without BMMSC or ACS therapy. Left ventricle was analyzed 8 h, 24 h or 72 h following CLP procedure. **A–C** mRNA expression of *Tlr-2* in fold change. **D–F** mRNA expression of *Tlr-4* in fold change. **G–I** mRNA expression of *Tlr-9* in fold change. Data are presented as mean ± SEM. p ≤ 0.05 was considered as statistically significant. *p ≤ 0.05, ** p ≤ 0.005, *** p ≤ 0.0005, CLP vs. sham, CLP vs. BMMSC, CLP vs. ASC. Each bar N = 5

A differential expression of pattern recognition receptors (PRR) involved in polymicrobial sepsis defense was found in the left ventricle after CLP with *Tlr2* expression upregulated at 8 h and 24 h (Fig. 6 A), which seemed to be reversed after both BMMSC and ASC treatment during the first 8 h after CLP procedure (Fig. 6A).

24h after sepsis induction, the expression of *Tlr4* was also upregulated in CLP. *Tlr9* was not upregulated in the left ventricle of CLP compared to Sham at any timepoints.

By contrast, *Tlr9* was upregulated in ASC in the 24 h observation period (Fig. 6H). Also, both ASCs and BMMSCs significantly increased *Tlr4* (Fig. 6F) expression, in the ASC group within 24 h after CLP treatment (Fig. 6E), in the BMMSC group within 72 h after CLP treatment.

### *Nlrp3* expression in left ventricle of CLP mice ameliorated after ASC administration

**Fig. 7:**
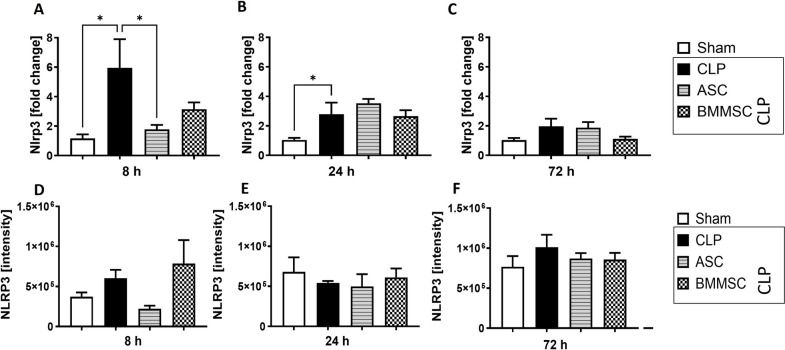
Upregulated NOD-, LRR- and pyrin domain-containing protein 3 (*Nlrp3*) expression in left ventricle of CLP mice. Wildtype mice received either sham treatment, CLP procedure with or without BMMSC or ACS therapy. Left ventricle was analyzed 8 h, 24 h or 72 h following CLP procedure. **A–C** mRNA expression of *Nlrp3* inflammasome in fold change and **D–F** protein expression in protein band intensity. Data are presented as mean ± SEM. p ≤ 0.05 was considered as statistically significant. *p ≤ 0.05, CLP vs. sham, CLP vs. BMMSC, CLP vs. ASC. Each bar N = 5

The Nlrp3 expression was assessed in CLP mice as another type of PRR and showed upregulation after 8 and 24 h (Fig. 7A, B). No changes in NLRP3 protein expression were observed (Fig. 7D–F). By contrast, Nlrp3 expression was significantly reduced by ASC administration in the 8 h treatment group (Fig. 7A), however not in the later stages. The effect by BMMSC administration appeared to be not significant.

## Discussion

The aim of the present study was to investigate the therapeutic effects of MSCs treatment on septic cardiomyopathy in the CLP mouse model. In particular, we focused on the immunomodulatory effects of the cells on the left ventricular tissue and compared the effects of two different types of MSCs—BMMSCs and ASCs.

We [13, 16, 44] and others [45, 46] have previously shown that exposure of mice to polymicrobial peritonitis during the CLP procedure induces elevation of systemic markers of cardiac injury (troponin I [47]) as well as local myocardial damage. In the present study, we confirmed these findings and showed that CLP treatment resulted in significant elevation of both systemic troponin I and HFABP at the early time point of 8 h. Interestingly, both MSCs reduced, although not significant, the elevation of troponin I and HFABP in septic mice.

Furthermore, we showed that polymicrobial sepsis induced morphological changes such as pyknosis, intramuscular hemorrhage, and ventricular rupture in all three polymicrobial sepsis groups which correlates with previously published findings of the depression of echo Doppler parameters of cardiac performance, such as cardiac output or left ventricular end diastolic pressure in the CLP model [13, 44].

At the molecular level, we confirmed the increased expression of *Tnfa*, *C3ar* and *C5ar1* as well as interleukins (*Il-1b*, *Il-6, Il-10*) in murine left ventricle in CLP animals. While the aforementioned upregulated cytokines and cytokine receptors have been shown to be crucial for the innate host response to polymicrobial sepsis, the complement receptors C3aR and C5aR have been shown to play a key role in the development of septic cardiomyopathy [11]. Three separate, but convergent pathways of complement activation led to increased levels of cleavage products C3a and C5a. The target receptor C3aR is shown to be significantly upregulated in human peripheral blood mononuclear cells during sepsis and is considered to be a key mediator in the early production of proinflammatory mediators such as IL-6 or TNFa and the severity of disease outcome during endotoxemic sepsis [48]. Indeed, 24 h after CLP treatment, the *C3ar* gene and C3a protein expressions peaked in murine left ventricle. However, some findings suggested that C3aR has a rather tempering role in the immune response during sepsis and its activation is protective against gram-negative bacteremia [49].

In contrast, elevated levels of C5a have been associated with a worse outcome in CLP-sepsis [50], and blocking the corresponding receptor C5aR with antibodies has been shown to be protective during sepsis [51]. Similarly, sepsis-induced cardiovascular dysfunction is reversed in mice lacking the C5aR [44]. The possible mechanism could be the previously shown disruption of the intracellular Ca^2+^ homeostasis by C5a exposure, resulting in impaired contractility [44, 52], release of extracellular histones [16] and cytoskeletal reorganization [53]. Furthermore, previous findings suggested a C5a/C5aR-dependent activation of the mitogen-activated protein kinase (MAPKs) pathway leading to cardiac dysfunction, which could be reversed by administration of a selective p38 MAPKs inhibitor [11]. Here, we confirmed the local increase of *C5ar1* expression in cardiomyocytes during sepsis as previously demonstrated [54], and showed a tendential downregulating effect of BMMSC administration. A similar effect of BMMSCs on C5ar expression could be demonstrated for renal tissue after ischemia–reperfusion injury via suppression of the C5a/C5aR-NF-κB pathway activation [55].

However, the stronger suppressive effect of BMMSCs was observed on C3aR in terms of gene expression. While the role of C3aR in septic cardiomyopathy is not fully understood, it has been associated with inflammatory cardiomyopathy [56] and fibrotic remodeling [57]. This in turn has been described in sepsis-induced myocardial dysfunction via Transforming Growth Factor β/Mothers against decapentaplegic homolog 3 (TGF-b/Smad3) signaling activation [58]. Both C3a and TGF-b can act as chemoattractants for MSCs to sites of inflammation or tissue remodeling [59–62]. MSCs themselves also possess C3aR, which upon C3a activation can lead to prolonged Extracellular-signal Regulated Kinases (ERK) 1/2 phosphorylation [59]. Interestingly, an in vitro study using the murine cardiac HL-1 cell-line, showed that ERK1/2 phosphorylation was promoted upon exposure to umbilical cord-derived MSCs preconditioned medium [63], suggesting a possible paracrine mechanism for cardiomyocytes. Although the murine *C3ar* gene has a rather small promoter site [64], it can be speculated whether the observed reduced *C3ar* gene expression in left ventricle of BMMSC-treated septic mice is an effect of target molecules activated by prolonged ERK1/2 phosphorylation. However, further downstream analyses are required to evaluate these mechanisms in detail.

Interestingly, we found that left ventricular *C5ar1* and *C3ar* gene expression peaked at 24 h while the expression of *Il-1b*, *Il-6* and *Tnfa* increased 8 h after CLP procedure. These results contrast with an in vitro study in which administration of C5a induced the release of these cytokines in rat cardiomyocytes [54].

The findings of local upregulation of *Il-1b, Il-6* and *Tnfa* gene expression are consistent with clinical data from sera of septic patients and their cardio-suppressive effect has been reproduced in vitro [65–68]. Il-10 has a more protective effect during polymicrobial sepsis and has been shown to improve the survival of CLP-mice [69, 70]. Notably, we found that after CLP procedure *Il-10* expression peaked later (24 h), than the expression of pro-inflammatory cytokines *Il-1b* and *Il-6.* In addition to a significant reduction of *Il-6* expression via ASCs, both MSC treatments resulted in a significant downregulation of the anti-inflammatory Il-10 24 h after CLP treatment, although the suppressive effect was stronger in BMMSC treatment. Up to our knowledge, the stronger effect on the gene expression of the pro-inflammatory cytokines IL-1b and IL-6 through ASCs compared to BMMSCs at the early (8 h) onset of sepsis is a new finding. This could partly be explained by recently described higher adherence ability of ASCs compared to BMMSCs [71], however, for the complement system we found a more significant effect of BMMSCs as previously described. Although our analyzed expression differences between BMMSC and ASC are limited to left ventricular tissue, it is interesting to note that while the majority of interleukins are produced by leucocytes such as monocytes, T-cells or macrophages [72], the majority of complement compounds is produced by hepatocytes[73]. Yet, we found expression differences between BMMSC and ASC in another group of inflammation mediators.

While the above-mentioned cytokines represent a pro-inflammatory response to sepsis, some TLRs play an important role in the defense against gram-negative bacteria, which constitute a large part of the gut microbiome [74]. Since the polymicrobial sepsis in our CLP model was caused by bacterial peritonitis, we additionally focused on the expression of TLRs. TLRs, as well as NLRP3, are PRRs and can increase cytokine production upon binding to the respective pathogen. TLR signaling and activation of the NOD, LRR-, and NLRP3 inflammasome have been previously associated with cardiac, hepatic, renal, and pulmonary injury in CLP-induced sepsis models in vivo [13], 75, 14, 76. The TLR -mediated inflammatory response has been shown to be mediated by C5a and C3a receptors, resulting in increased MAPK and nuclear factor-κB (NF-κB) activation [77]. Notably, C5aR appears to play a predominant role in TLR4 signaling, whereas C3aR plays a role in TLR9 signaling [77]. In our study, gene expression of both, *C5ar1* and *Tlr4* peaked 24 h after CLP treatment and remained upregulated at 72 h. Such dynamic was not observed for *C3ar,* which could be explained by the absence of *Tlr9* upregulation in the CLP group. The respective persistent upregulation of *C5ar* and *Tlr4* at a late time point may result from mutual maintenance, since TLR-induced inflammatory cytokines can upregulate the expression of C5aR and C3aR [77]. However, in our case, the increased expression of the corresponding cytokines of *Il-6, Tnfa*, and *Il-1b* occurred at an earlier timepoint. Although ASC administration may downregulate Il-6 expression in CLP mice at an earlier time point, it has an opposite effect on *Tlr4* and *Tlr9* at a later time point. Upregulated *Tlr4* in the ASC treated group of the 72 h observation point as well as in the earlier 24 h point in the BMMSC group is an interesting finding for several reasons. First, TLR-4 is expressed in both BMMSCs [78] and ASCs [79, 80], which might enable a paracrine interaction between downstream pathways of cardiac and MSC-expressed TLR-4. Activation of TLR-4 may increase production of pro-inflammatory cytokines such as IL-6 [81], which in turn has been shown to induce Tlr4 gene expression in human skeletal muscle via Signal transducer and activator of transcription 3 (STAT3) [82]. Moreover, it has been shown that MSCs with depleted TLR-4 improved cardiac output of rats with ischemic heart injury upon systemic application more effective compared to rats which received TLR-4-expressing MSCs – interestingly also via STAT3 activation [83].

These results might suggest a TLR-4 dependent harmful effect of MSCs on the heart, however, the above-mentioned study also showed an improved cardiac function of rats with ischemic cardiac injury which received WT-MSCs compared to untreated rats, even if the effect was slimmer[83]. Also, measurements of cardiac functions were not performed in our study, which makes the results difficult to compare. Noteworthy, we could also not detect a significant increase of MyD88 protein expression in BMMSC or ASC (supplemental Fig. 2). MyD88 is an adapter protein, which connects with the cytoplasmic part of most TLRs—including TLR-4—to recruit IL-1R-associated kinase 4 (IRAK-4) and IRAK-1 through a homophilic interaction [84]. However, it should be noted that particularly TLR-4 can be activated in a MyD88-independent pathway as well [85]. To determine whether increased left ventricular Tlr4 expression in BMMSC starting 24 h after CLP procedure or in ASC at the 72 h observation point have more cardioprotective or inflammation-maintaining effects, more studies on the downstream TLR-4 pathways of both MSCs and cardiac cells would be necessary. The lack of significant changes in local *Tlr9* expression induced by bacterial peritonitis and the concomitant changes in local expression of C3ar support the idea that host-regulated immunological damage can occur without the local presence of pathogens [84]. Peculiarly, *Tlr9* expression is upregulated in left ventricular tissue of the ASC group at 24 h while the *Nlrp3* expression is downregulated earlier at 8 h, since the it has been shown that TLR-9 activates the NLRP3 inflammasome in a murine macrophage cell line [86].

Like the TLR, the NLRP3 inflammasome is also a PRR and can be activated by intravenous injection of LPS, which can lead to the release of IL-1b and TNFa [87]. In a previous study in mice, our group showed that during CLP sepsis, the NLRP3 inflammasome may mediate the systemic elevation of extracellular histones in plasma, which was associated with apoptosis, mitochondrial dysfunction, and increases in cytosolic reactive oxygen species (ROS) and intracellular calcium [Ca2 +] in cardiomyocytes [13]. Here, we demonstrated that *Nlrp3* expression in CLP mice is upregulated at early stage and persists up to 24 h, and that ASC administration reduces it at the 8 h time point.

We were also able to show that intravenous application of MSCs reduced the expression of *Il-6* and *C3ar*, *Nlrp3* and *Tlr2* in cardiac tissue during earlier stages, but not at the 72 h observation period. Those findings did not reflect in our histological analysis of left ventricular tissue. Comparing ASCs and BMMSCs, our data shows that ASCs have a stronger effect on *Il-6, Il-1b* and *Nlrp3* gene expression, whereas BMMSCs have a stronger effect on complement receptor expression. Previous studies comparing the therapeutic effects of BMMSC and ASC in vivo did not find differences between these two types of MSCs [34], which could be explained by differences in study designs and measurements.

## Conclusion

In conclusion, we demonstrated that polymicrobial sepsis in mice induced cardiac damage via systemic elevation of cardiac damage markers and local elevation of pro-inflammatory cytokines and septic cardiomyopathy associated PRR.

MSC treatment transiently decreased the expression of *C3ar, Tlr2, Il-10 Il-1b*, *Il-6* and *Nlrp3* in the left ventricle and may therefore have transient anti-inflammatory therapeutic effects during the early observation period of up to 24 h. Of the two different MSC sources, bone marrow-derived MSC had a greater effect on the complement system, whereas adipose-derived MSC had a stronger effect on the expression of *Nlrp3, Il-6* and *Il-1b* genes.

## Supplementary Information

Below is the link to the electronic supplementary material.

Supplementary file1 (DOCX 14 KB)

## Data Availability

Data is provided within the manuscript or supplementary information files.

## Abbreviations

ASC: Adipose stromal cells
BMMSC: Bone-marrow derived mesenchymal stem cells
C3ar: Complement component 3a receptor
C5ar1: Complement component 5a receptor 1
CLP: Cecal ligation puncture procedure
DAMP: Damage-associated molecular pattern
ELISA: Enzyme-Linked Immunosorbent Assay
ERK: Extracellular-signal regulated kinases
HFABP: Heart-type fatty acid binding protein
ICU: Intensive care unit
Il-10: Interleukin-10
Il-1b: Interleukin-1β
Il-6: Interleukin-6
I.P.: Intraperitioneally
LRR: Leucine rich repeat
MAP: Mitogen-activated protein kinase
MSC: Mesenchymal stem cell
MyD88: Myeloid differentiation primary-response protein 88
Nlrp3: NOD-, LRR- and pyrin domain-containing protein 3
NOD: Nucleotide-binding oligomerization domain-like receptors
TGFB: Transforming growth factor β
QPCR: Quantitative reverse transcription polymerase chain reaction
Tlr2: Toll-like receptor 2
Tlr4: Toll-like receptor 4
Tlr9: Toll-like receptor 9
TNFA: Tumor necrosis factor alpha
TNI: Roponin I
PBS: Phosphate buffered saline
PFA: Paraformaldehyde
PL: Platelet lysate
ROS: Reactive oxygen species
S.C.: Subcutaneously
Smad3: Mothers against decapentaplegic homolog 3
STAT3: Signal transducer and activator of transcription 3
