# Supplementary material for: Immunomodulatory and cardio-protective effects of differentially originated multipotent mesenchymal stroma cells during polymicrobial sepsis in mice
Source: Eur J Trauma Emerg Surg. 2025 Apr 20;51(1):178. doi: 10.1007/s00068-025-02862-2 (PMC12009780; doi:10.1007/s00068-025-02862-2)
Supplement: Supplementary file 1 — Supplementary file1 (DOCX 14 KB) [file 68_2025_2862_MOESM1_ESM.docx]

**Supplemental Figure 1: Sketch of the sample collection from the murine heart.** Immediately after explantation, the heart was separated into right (RV) and left ventricle (LV). The upper part of LV (1) was conserved in 4% PFA for histological sections, (2), (3) and RV were conserved in liquid nitrogen. Part (2) was used for Western Blot and (3) for RNA extraction.

**Supplemental Figure 2: No significant changes of the myeloid differentiation primary-response protein 88 (MyD88) in left ventricle of CLP mice** Wildtype mice received either sham treatment, CLP procedure with or without BMMSC or ACS therapy. Left ventricle was analyzed 8 h, 24 h or 72 h following CLP procedure. **(A-C)** protein expression of MyD88 in protein band intensity. Data are presented as mean ± SEM. p ≤ 0.05 was considered as statistically significant. *p ≤ 0.05, CLP vs. sham, CLP vs. BMMSC, CLP vs. ASC. Each bar N=5.
